# Blood Pressure Control and Cardiovascular and Mortality Risk in VA Nursing Home Residents

**DOI:** 10.1093/geroni/igab046.2320

**Published:** 2021-12-17

**Authors:** Xiaojuan Liu, Sei Lee, Michael Steinman, Laura Graham, Yongmei Li, Bocheng Jing, Michelle Odden

**Affiliations:** 1 Stanford University, Stanford, California, United States; 2 University of California San Francisco, San Francisco, California, United States; 3 VA Palo Alto Health Care System, Menlo Park, California, United States; 4 San Francisco VA Medical Center, San Francisco, California, United States

## Abstract

Optimal blood pressure (BP) control in nursing home residents is controversial and this population has been excluded from trials. We evaluated the associations of BP level with cardiovascular (CV) events and all-cause mortality across antihypertensive medication categories in Veterans Affairs (VA) nursing home residents. Data for 18,589 residents aged 65 years and older was obtained from the VA Corporate Data Warehouse from October 2006 through September 2017. Baseline systolic BP (SBP) and diastolic BP (DBP) were divided into categories and analyses were stratified by antihypertensive therapy (0, 1, and ≥2 medications). Over a median follow-up of 1.8 years, CV events occurred in 3,519 (19%) residents and 15,897 (86%) residents died. In participants on no BP medications, high SBP (>150 mmHg) was associated with a greater risk of CV events (adjusted [cause-specific] hazard ratio, 1.39; 95% confidence interval, 0.94-2.06) compared with normal SBP (110-130mmHg). By contrast, in participants on ≥2 BP medications, the subgroup with low SBP (<110 mmHg) had a higher CV risk (1.38; 1.20-1.57). For DBP, in participants without BP medications, there were no differences in CV risk across DBP subgroups. Whereas among those on 1 or ≥2 medications, DBP <60 mmHg was associated with a higher CV risk (1.26; 1.03-1.55 and 1.35; 1.18-1.54, respectively) compared with normal DBP (70-80 mmHg). Participants with low SBP (<110 mmHg) and DBP (<70 mmHg) had an increased mortality risk regardless of the number of medications. These findings suggest a potential risk of low BP among nursing home residents on multiple antihypertensive medications.

